# Supplementary material for: The Effect of an Electronic Passive Prompt Intervention on Prolonged Occupational Sitting and Light-Intensity Physical Activity in Desk-Based Adults Working from Home during COVID-19 in Ireland
Source: Int J Environ Res Public Health. 2023 Jul 4;20(13):6294. doi: 10.3390/ijerph20136294 (PMC10341828; doi:10.3390/ijerph20136294)
Supplement: Supplementary file 1 [file ijerph-20-06294-s001.zip › ijerph-2462280-supplementary.pdf]

Supplementary Data File:

Journal Title: The Effect of an Electronic Passive Prompt Intervention on Prolonged Occupational Sitting and Light-Intensity Physical Activity in Desk-Based Adults Working from Home During COVID-19 in Ireland

Authors: Aidan J. Buffey [1,2,*], Gráinne Hayes [1,2], Brian P. Carson [1,2,†] and Alan E. Donnelly [1,2,†]

Affiliations:

[1] Department of Physical Education and Sport Science, University of Limerick Faculty of Education and Health Sciences, Limerick, Ireland

[2] Physical Activity for Health Research Cluster, Health Research Institute (HRI), University of Limerick, Limerick, Ireland

[#] Denotes that both authors contributed equally and are joint senior authors.

[*] Correspondence: aidan.buffey@ul.ie

Supplementary Table S1. This table presents the sedentary behaviour patterns during the 24-hour period throughout the baseline (Week 1-3) and intervention (Week 4-6). Presented as Mean ± Standard Error (95% Confidence Intervals).

Total 24-Hour Sedentary Behaviours Patterns	Baseline	Intervention	Mean Change	*p*	*d*
**Number of SB > 40 (Mins)**	**4.84 ± 0.27 (4.30-5.39)**	**4.50 ± 0.29 (3.91-5.09)**	**-0.34 ± 0.15 (-0.60--0.05)**	**0.026**	**0.171**
**Time in SB > 40 (Mins)**	**368.86 ± 25.60 (316.33-421.38)**	**324.20 ± 25.27 (272.34-376.05)**	**-44.66 ± 11.95 (-69.17--20.14)**	**< 0.001**	**0.341**
**Number of SB > 60 (Mins)**	**2.66 ± 0.22 (2.20-3.11)**	**2.24 ± 0.21 (1.82-2.66)**	**-0.42 ± 0.14 (-0.70--0.14)**	**0.005**	**0.254**
**Time in SB > 60 (Mins)**	**261.27 ± 24.52 (210.96-311.57)**	**214.607 ± 22.40 (168.65-260.56)**	**-46.67 ± 13.74 (-74.85--18.47)**	**0.002**	**0.299**
**Number of SB > 90 (Mins)**	**1.21 ± 0.14 (0.92-1.50)**	**0.96 ± 0.12 (0.71-1.22)**	**-0.25 ± 0.08 (-0.41--0.09)**	**0.004**	**0.265**
**Time in SB > 90 (Mins)**	**155.97 ± 19.93 (115.07-196.86)**	**121.80 ± 16.94 (87.05-156.56)**	**-34.16 ± 11.28 (-57.31--11.02)**	**0.005**	**0.254**
Total Number of SB	47.03 ± 3.05 (40.76-53.29)	48.43 ± 3.15 (41.97-54.90)	+1.41 ± 1.07 (-0.79-3.60)	0.200	0.060
**Total Time Spent in SB (Mins)**	**672.56 ± 18.62 (634.37-710.76)**	**640.44 ± 17.02 (605.52-675.36)**	**-32.13 ± 9.35 (-51.30--12.95)**	**0.002**	**0.304**
**Total Sedentary Waking Hours**	**11.21 ± 0.31 (10.57-11.85)**	**10.67 ± 0.28 (10.57-11.85)**	**-0.54 ± 0.16 (-0.86--0.22)**	**0.002**	**0.305**

Abbreviations: SB; sedentary bouts. **Bold** indicates that a significant change was found for the reported outcome variable ($p < 0.05$).

Supplementary Table S2. This table presents the sedentary behaviour patterns during the non-work (leisure) hours throughout the baseline (Week 1-3) and intervention (Week 4-6). Presented as Mean ± Standard Error (95% Confidence Intervals).

Leisure Sedentary Behaviours Patterns	Baseline	Intervention	Mean Change	*p*	*d*
Number of SB > 40 (Mins)	1.91 ± 0.14 (1.61-2.20)	1.71 ± 0.16 (1.38-2.04)	-0.19 ± 0.11 (-0.42-0.04)	0.102	0.096
Time in SB > 40 (Mins)	141.97 ± 12.53 (116.25-167.68)	126.55 ± 13.00 (99.87-153.24)	-15.41 ± 8.97 (-33.81-2.99)	0.097	0.099
**Number of SB > 60 (Mins)**	**1.02 ± 0.10 (0.82-1.22)**	**0.86 ± 0.10 (0.66-1.06)**	**-0.15 ± 0.07 (-0.29--0.01)**	**0.032**	**0.159**
Time in SB > 60 (Mins)	98.38 ± 11.18 (75.44-121.33)	84.91 ± 10.61 (63.13-106.69)	-13.47 ± 7.35 (-28.54-1.60)	0.078	0.111
Number of SB > 90 (Mins)	0.43 ± 0.06 (0.30-0.57)	0.41 ± 0.07 (0.28-0.55)	-0.02 ± 0.05 (-0.13-0.09)	0.681	0.006
Time in SB > 90 (Mins)	55.72 ± 9.50 (36.23-75.20)	51.92 ± 8.89 (33.68-70.16)	-3.80 ± 7.38 (-18.93-11.34)	0.611	0.010
Total Number of SB	22.84 ± 1.58 (19.60-26.08)	23.24 ± 1.54 (20.09-26.39)	+0.40 ± 0.63 (-0.89-1.69)	0.528	0.015
Total Time Spent in SB (Mins)	289.51 ± 10.53 (267.91-311.12)	275.09 ± 12.27 (249.91-300.27)	-14.42 ± 8.99 (-32.88-4.03)	0.120	0.087
Total Sedentary Leisure Waking Hours	4.83 ± 0.18 (4.47-5.19)	4.58 ± 0.21 (4.17-5.00)	-0.24 ± 0.15 (-0.55-0.07)	0.119	0.087

Abbreviations: SB; sedentary bouts. **Bold** indicates that a significant change was found for the reported outcome variable ($p < 0.05$).
